# Supplementary material for: Switchable photovoltaic windows enabled by reversible photothermal complex dissociation from methylammonium lead iodide
Source: Nat Commun. 2017 Nov 23;8:1722. doi: 10.1038/s41467-017-01842-4 (PMC5701074; doi:10.1038/s41467-017-01842-4)
Supplement: Supplementary file 1 — Supplementary Information [file 41467_2017_1842_MOESM1_ESM.pdf]

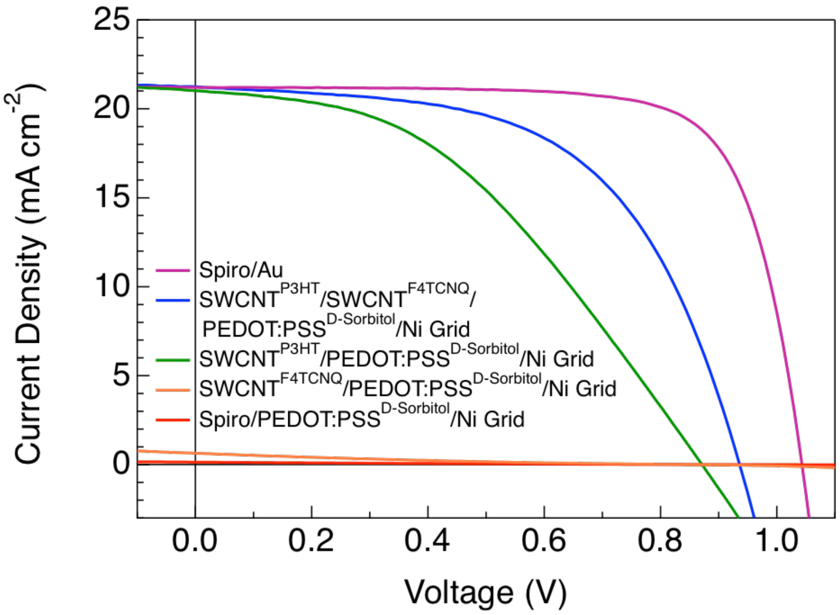

Supplementary Figure 1. Comparison of current-voltage characteristics for various PV device hole transport architectures. PV parameters extracted from this data are summarized in Supplementary Table 1.

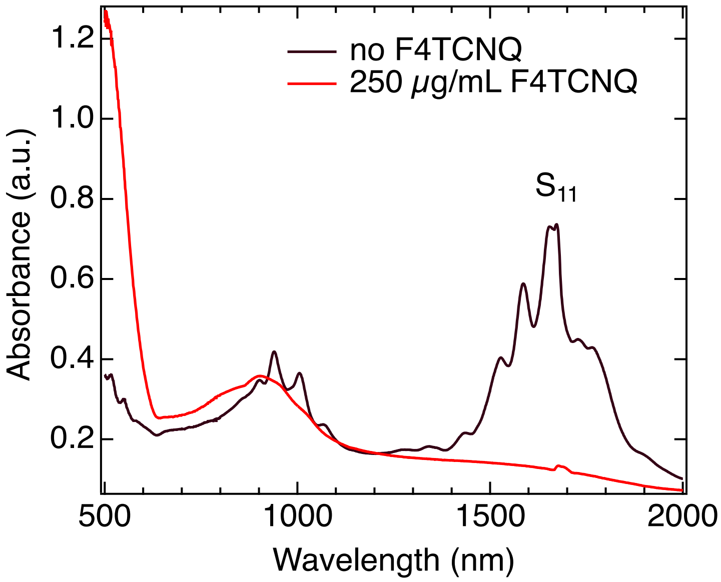

Supplementary Figure 2. Optical absorption spectra of ink used to generate SWCNT^{F4TCNQ} layer. A SWCNT/PFPD ink (black spectrum) was doped in the solution phase with the charge transfer dopant F4TCNQ at a doping concentration of 250 μg mL^{-1} (red spectrum). Adding F4TCNQ to the SWCNT ink results in bleaching of the S_{11} transitions, indicative of a charge transfer interaction between the dopant and SWCNTs in the solution phase. The spectrum in red displays the ink that was sprayed directly onto the SWCNT/P3HT layer in the device stack.

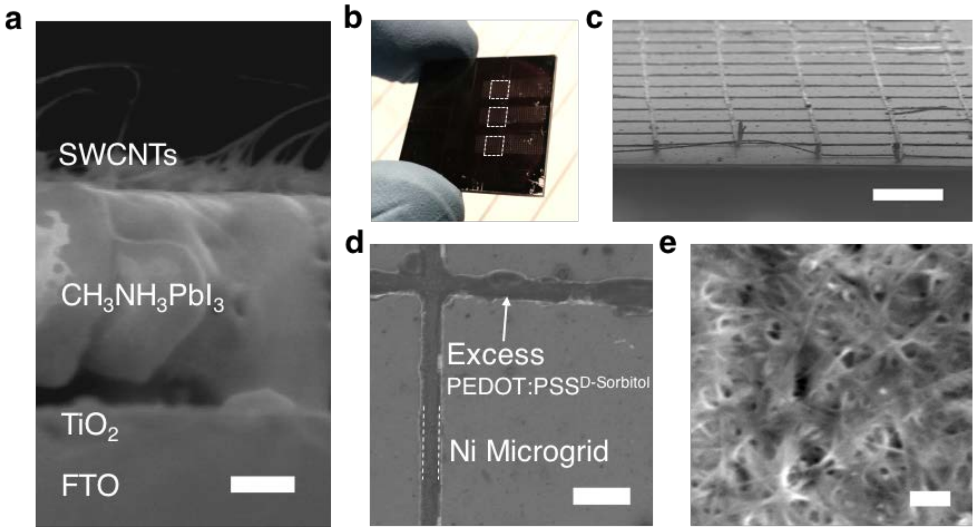

Supplementary Figure 3. Microscopy Characterization of Switchable PV Devices. (**A**) SEM cross-section image of device showing the layers beneath the Ni grid coated with PEDDOT:PSS$^{D\text{-}Sorbitol}$. Scale bar is 200 nm (**B**) Photograph of device with three complete pixels highlight with dashed white lines. (**C**) SEM image highlighting the Ni grid top contact. Scale bar is 300 µm (**D**) SEM image of Ni grid coat (white lines) coated with PEDDOT:PSS$^{D\text{-}Sorbitol}$ laminated onto the SWCNT^{F4TCNQ} layer. Excess PEDDOT:PSS$^{D\text{-}Sorbitol}$ is visible along the grid. Scale bar is 50 µm. (**E**) SEM image showing the porous network of SWCNT^{F4TCNQ} that allows gas to permeate through to the $CH_3NH_3PbI_3$ layer for switching.

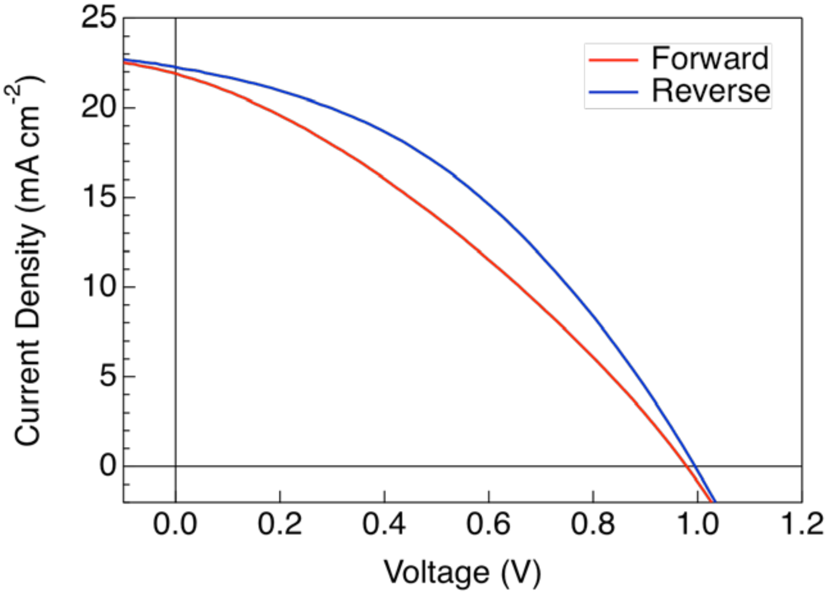

Supplementary Figure 4. Hysteresis of forward and reverse scans of as-produced switchable PV cell. The device was stored in a N_2-filled glovebox for three months before the measurement.

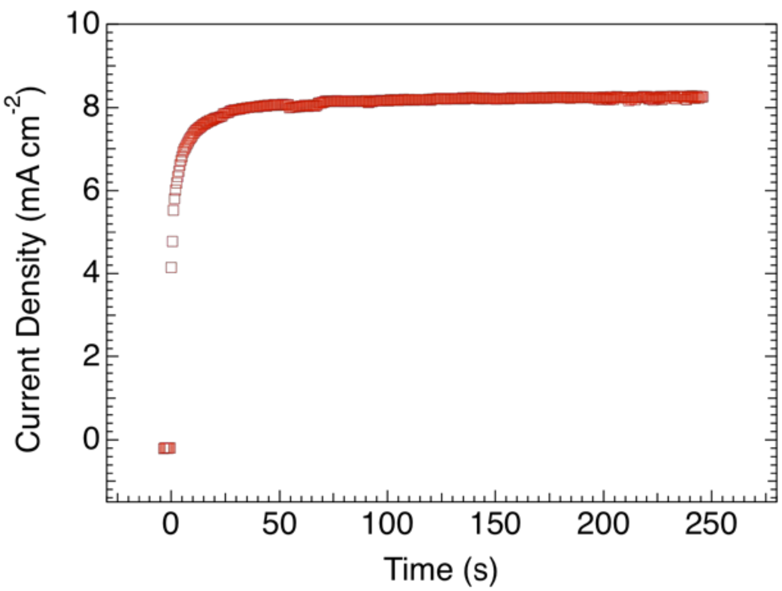

Supplementary Figure 5. Stabilized power output of as-produced switchable PV cell. The device was stored in a N_2-filled glovebox for three months before the measurement.

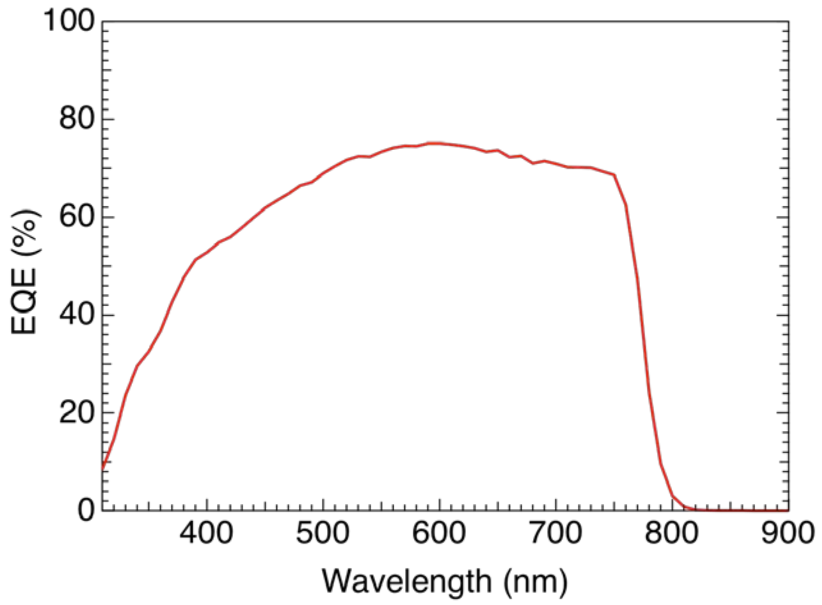

Supplementary Figure 6. **External Quantum Efficiency of as-produced switchable PV cell.** The device was stored in a N_2-filled glovebox for three months before the measurement.

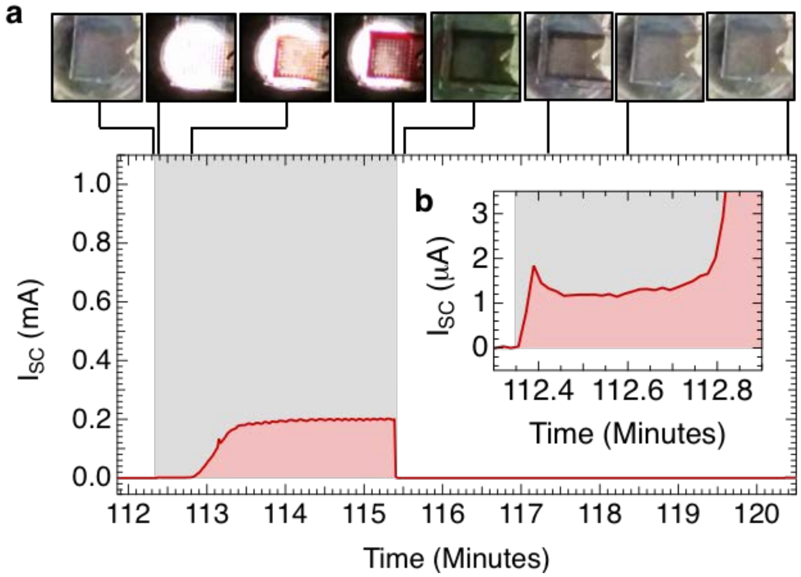

Supplementary Figure 7. Current dynamics of fifteenth light-dark cycle. (**A**) Short-circuit current as a function of time for the fifteen light-dark cycle shown in Fig. 1 with corresponding still-frames showing continued device switching. (**B**) Short-circuit current at early times to show similar kinetics of first cycle shown in Fig. 1.

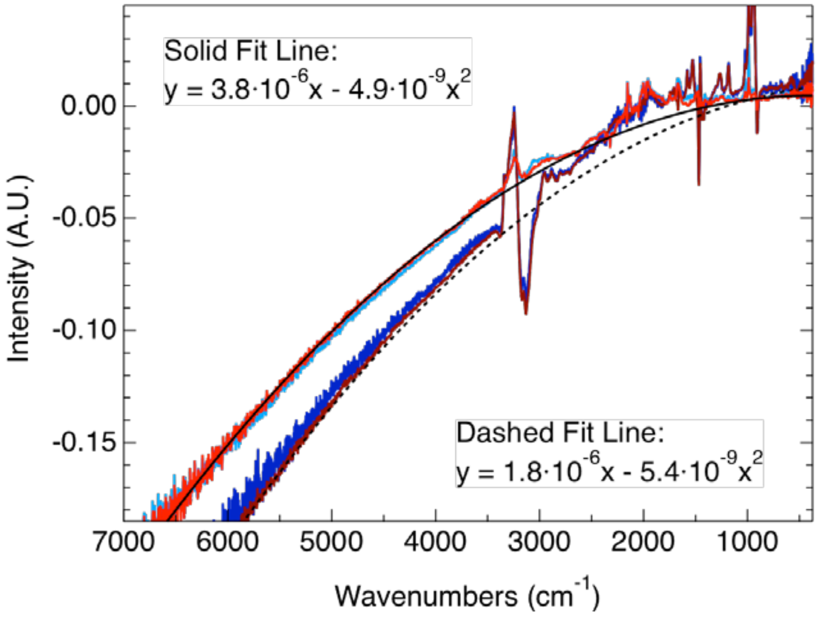

Supplementary Figure 8. Background subtraction from FTIR data. The color of the spectra corresponds to Fig. 2. The solid line is a 3rd order polynomial fit to the data at 60 °C. The dashed line is a 3rd order polynomial fit to the data at 25 °C. The fit parameters are shown as insets.

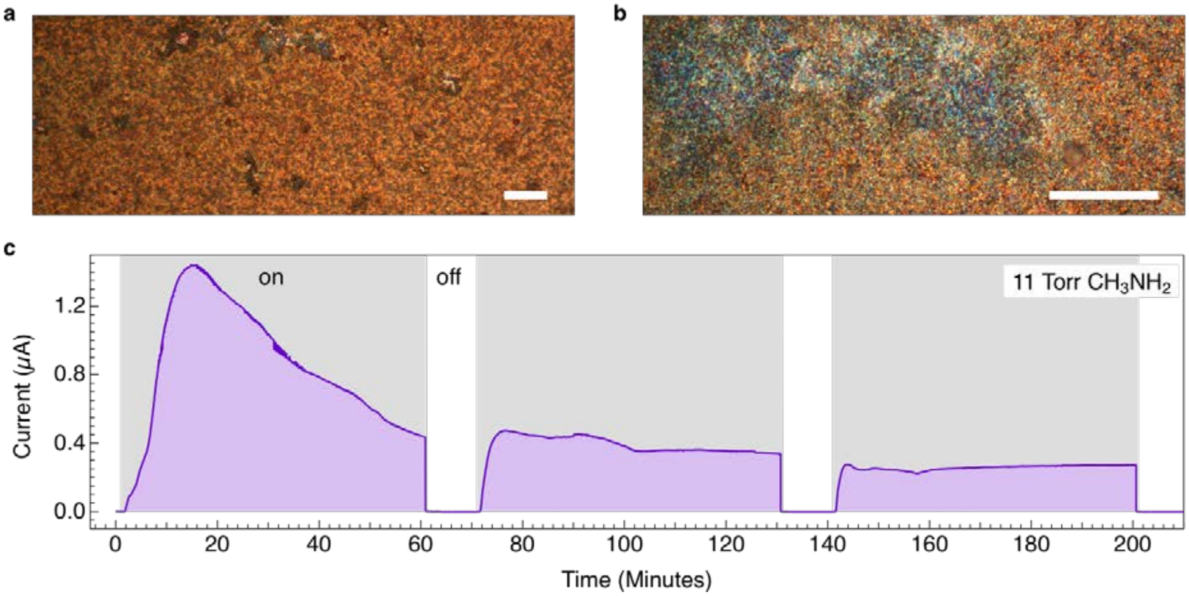

Supplementary Figure 9. Optical microscopy and current dynamics of switchable photoresistor. Low (**A**) and high (**B**) magnification of optical microscope images after 3 1-hour illumination cycles. Scale bars are 50 μm. (**C**) Current as a function of time for a $CH_3NH_3PbI_3$•$x$$CH_3NH_2$ photoresisitor with 11 Torr CH_3NH_2. The device was illumintated for 1 hour and allowed to cool for 10 minutes.

Supplementary Table 1. PV Device performance for various hole transport architectures

Hole Transport Architecture	Visibly Transparent	Vapor Permeable	V_{OC} (V)	J_{SC} (mA cm^{-2})	FF (%)	PCE (%)
$SWCNT^{F4TCNQ}$/ $PEDOT:PSS^{D\text{-}Sorbitol}$/Ni Grid	Yes	Yes	0.71 ± 0.24	0.6 ± 0.1	0.19 ± 0.04	0.08 ± 0.02
SWCNT/P3HT/ $PEDOT:PSS^{D\text{-}Sorbitol}$/Ni Grid	Yes	Yes	0.87 ± 0.01	20.7 ± 0.5	0.34 ± 0.06	6.2 ± 1.1
SWCNT/P3HT/$SWCNT^{F4TCNQ}$/$PEDOT:PSS^{D\text{-}Sorbitol}$/Ni Grid	Yes	Yes	0.93 ± 0.01	20.6 ± 0.7	0.54 ± 0.03	10.3 ± 0.9
Spiro-OMeTAD/ $PEDOT:PSS^{D\text{-}Sorbitol}$/Ni Grid*	Yes	Yes	0.96 ± 0.04	0.2 ± 0.1	0.20 ± 0.01	0.04 ± 0.01
Spiro-OMeTAD/Au	No	No	1.05 ± 0.01	21.2 ± 0.3	0.73 ± 0.01	16.3 ± 0.1

Supplementary Table 2. Chemical composition XPS analysis. Chemical composition determined by analysis of the C 1s, N 1s, O 1s, I 4d, and Pb 4f XPS peaks for photoresistors in three different conditions. Uncertainty of reported value is ±5%

Condition	C (%)	N (%)	O (%)	I (%)	Pb (%)	I:Pb	I:N	Pb:N
Control (0 cycles)	63.39	7.55	0.8	19.98	8.28	2.41	2.65	1.10
0 Torr CH_3NH_2, 20 cycles	40.43	8.97	2.86	33.12	14.63	2.26	3.69	1.63
11 Torr CH_3NH_2, 20 cycles	28.85	12.54	2.5	39.43	16.67	2.37	3.14	1.33
