# Supplementary material for: Switchable photovoltaic windows enabled by reversible photothermal complex dissociation from methylammonium lead iodide
Source: Nat Commun. 2017 Nov 23;8:1722. doi: 10.1038/s41467-017-01842-4 (PMC5701074; doi:10.1038/s41467-017-01842-4)
Supplement: Supplementary file 3 — Description of Additional Supplementary File [file 41467_2017_1842_MOESM3_ESM.pdf]

Description of Additional Supplementary File

File Name: Supplementary Movie 1
Description: Movie showing the first illumination cycle of a switchable PV device described in Figure 1e. Time is sped up for durations of the movie as indicated by the multiplier in the bottom right.
